# Ubiquitin‐independent, Proteasome‐mediated targeted degradation of KRAS in pancreatic adenocarcinoma cells using an engineered ornithine decarboxylase/antizyme system

**DOI:** 10.1002/iub.1945

**Published:** 2018-10-22

**Authors:** Yihui Ma, Jingjing Xu, Pei Huang, Xue Bai, Hanqing Gao

**Affiliations:** ^1^ Department of Pathology Zhengzhou University 1st Affiliated Hospital, Zhengzhou China

**Keywords:** ornithine decarboxylase, antizyme, KRAS, pancreatic adenocarcinoma, proteasome, protein degradation, gene therapy

## Abstract

The oncogene *KRAS* not only promotes the tumorigenesis of pancreatic cancers but also is required for the malignant progression and metastasis of these cancers. Many methods have been explored to influence the malignant biological behavior of these cancers by targeting mutant *KRAS*. The ornithine decarboxylase/antizyme (ODC/AZ) system is another protein degradation pathway that exists in nature. The formation of an ODC and protein substrate complex through direct combination can promote its degradation by the 26S proteasome without ubiquitination, and this process can be catalyzed by AZ. In this study, we designed and reconstructed a chimeric fusion protein (named RC‐ODC). The engineered fusion protein RC‐ODC was confirmed to interact with the mutant KRAS oncoprotein in a co‐immunoprecipitation assay, and the introduction of both RC‐ODC and AZ resulted in degradation of the exogenous and endogenous mutant KRAS oncoprotein at the post‐translational level independent of ubiquitination in vitro. Along with a decreased KRAS level, suppression of PANC‐1 cell proliferation was detected in vitro and in vivo, and meanwhile downregulation of phosphorylated extracellular signal‐regulated kinase 1/2 (ERK1/2) was also observed. Targeted degradation of the KRAS oncoprotein through the ODC/AZ pathway at the post‐translational level may reflect a more effective future therapeutic strategy for pancreatic cancer patients. © 2018 The Authors. IUBMB Life published by Wiley Periodicals,Inc. on behalf of International Union of Biochemistry and Molecular Biology, 71(1):57–65, 2019

**Abbreviations**
ASO: antisense deoxyoligonucleotides
AZ: antizyme
C: carboxyl
ERK: extracellular signal‐regulated kinase
G: glycine
HA: hemagglutinin
N: amino
ODC: ornithine decarboxylase
PROTACs: proteolysis‐targeting chimeric molecules
RNAi: RNA interference
RT‐PCR: real‐time reverse transcription PCR
S: serine
STR: short tandem repeat
UPP: ubiquitin proteasome pathway

## INTRODUCTION

The *Ras* gene family has three members, including *KRAS*, which has the greatest impact on human cancer 1. In a normal physiological state, *KRAS* encodes small GTPases to regulate cell differentiation, growth, and survival through signal transduction, whereas abnormal activation of mutant *KRAS* causes signal transduction disorder in cells and leads to the occurrence of multiple types of tumors 2, 3. *KRAS* mutation has been confirmed to be one of the earliest genetic changes in pancreatic carcinogenesis and has been observed in more than 90% of pancreatic cancers 4. Therefore, the silencing of mutant *KRAS* or direct elimination of the KRAS oncoprotein has been regarded as an efficient strategy for the targeted treatment of pancreatic cancers 5, 6, 7. With the development of molecular biology techniques, the *KRAS* gene has been silenced at the transcriptional level by several methods, such as antisense deoxyoligonucleotides (ASO), triple‐helix DNA, and RNA interference (RNAi). Among these, RNAi has been used successfully in experimental animal models of pancreatic cancer 8, 9, 10.

The ubiquitin proteasome pathway (UPP) is a “highly conserved pathway” for the irreversible elimination of protein substrates in cells and thus provides an important means through which cells can directly and effectively destroy misfolded/damaged proteins through ubiquitination 11, 12. Considering the high specificity and validity of substrate elimination through the UPP, this pathway has been used to investigate the degradation of abnormal protein substrates at the post‐translational level 13, 14, 15. A few reconstructed fusion E3 ligases and proteolysis‐targeting chimeric molecules (PROTACs) have been generated by successfully eliminating their corresponding protein substrates in cells, which can significantly influence the malignant characteristics of tumors in vitro and in vivo 16, 17, 18. Similarly, in our previous study, we also generated a “U‐box‐based” fusion E3 ligase named “RC‐U” that effectively interacted with, ubiquitinated, and promoted KRAS oncoprotein degradation at the post‐translational level. Importantly, the RC‐U fusion E3 ligase also successfully slowed down the proliferation of the pancreatic cancer cell lines PANC‐1 and MIAPaCa‐2 19.

However, formation of the engineered E3 ligase and protein substrate complex requires an accurate spatial geometric structure, which allows ubiquitin‐conjugating enzymes (E2s) to catalyze the addition of ubiquitin molecules onto specific lysine residues in the protein substrate 20, 21. The precise requirements for the structure indicate that valid ubiquitination of protein substrates may be difficult due to mismatched rigidity between protein substrates and E3 ligases 22, 23, 24. In fact, we attempted to design and reconstruct some chimeric fusion E3 proteins, and only RC‐U effectively interacted with, ubiquitinated, and promoted KRAS oncoprotein degradation, suggesting that successful ubiquitination of protein substrates reduces the efficiency of “degradation of proteins by the UPP system,” as well as restricts the possible applications to a certain extent 19.

Some of the difficulties in the valid ubiquitination of targeted protein substrates can be overcome through the use of another system named “ornithine decarboxylase (ODC) and antizyme (AZ)”. The ODC/AZ system was recently discovered and confirmed to be another pathway for proteasome‐dependent protein degradation that exists in nature (25, 26. ODC is a pivotal protein molecule that participates in the biosynthesis of various polyamines. AZ, which stands for “anti‐enzyme for ornithine decarboxylase”, is a regulatory protein of ODC and was initially verified as an important enzyme in polyamine biosynthesis 27, 28. The direct combination of ODC and AZ results in a change in the three‐dimensional structure of ODC that exposes the proteasome‐binding site in its carboxyl terminus 29, 30. Finally, ODC is eliminated through the proteasome. Obviously, this process does not rely on ubiquitination. Thus, the degradation of protein substrates by ODC/AZ might be more direct and effective compared with UPP.

In the present study, a novel engineered fusion protein named RC‐ODC that can connect the KRAS‐binding domain RBD + CRD of RAF‐1 with ODC was reconstructed to eliminate the targeted KRAS oncoprotein through the ODC/AZ pathway. In fact, our data confirmed that the chimeric RC‐ODC fusion protein molecule downregulated the level of the KRAS oncoprotein after co‐transfection with AZ and inhibited the growth of the pancreatic cancer cell line PANC‐1 in vitro and in vivo.

## EXPERIMENTAL PROCEDURES

### Cloning of cDNA and Construction of Expression Plasmids

The cDNA sequences encoding human AZ (70–228 aa) with a hemagglutinin (HA) tag, the full‐length ODC protein, and the RBD + CRD domain of RAF‐1 (named “RC” for short) were generated by PCR amplification of normal human lung tissues. The AZ and ODC cDNAs were incorporated into the upstream Nhe I/downstream BamH I and the upstream Not I/downstream Xho I sites of the pcDNA3.1 plasmid (Invitrogen) with HA and Myc tags, respectively, and named pcDNA3.1‐AZ‐HA and pcDNA3.1‐ODC‐Myc. The RC and ODC cDNA sequences were successively connected into the upstream BamH I site and the downstream Xho I site of the pcDNA3.1 plasmid and named pcDNA3.1‐RC‐ODC‐Myc. We used four glycine‐serine repeat sequences (GSGSGSGS) to link the RC to ODC sequences to avoid interference between the two domains. The nucleotide sequence encoding mutant *KRAS* ^G12D^ carrying the Flag tag was generated by PCR amplification of the cDNAs of PANC‐1 cells and was inserted into the BamH I/Xho I sites of the pcDNA3.1 plasmid 12. For lentivirus‐based fusion protein expression, we subcloned the AZ and RC‐ODC cDNAs into the pLenti6.3‐MSC‐IRES‐EGFP (Invitrogen) to generate pLenti‐AZ‐RFP and pLenti‐RC‐ODC‐GFP, respectively. Lentivirus packaging was carried out in human HEK 293T cells. Lastly, the viral titers were analyzed and determined.

### Cell Lines and Cell Culture

We purchased human HEK 293T cells and the human pancreatic cancer cell line PANC‐1 from the American Type Culture Collection. Cells were cultured in Dulbecco's Modified Eagle's Medium (Sigma‐Aldrich, Poole, UK) with 10% fetal bovine serum (GIBCO) at 37 °C in a 5% CO_2_ atmosphere. All cell lines were examined 2 weeks before the experiments by assessing their morphology under microscopy, performing a growth curve analysis and detecting mycoplasma in accordance with the ATCC cell line verification test recommendations.

### Cells Transfection and Infection

We used the Lipofectamine 2000 transfection reagent (Invitrogen) to perform the transient transfections of cells in 6‐well plates. The cells were transfected with the AZ‐, ODC‐, mKRAS‐, or RC‐ODC‐expressing plasmids according to the requirements of the product instructions. For stable expression, 7 × 10^4^ cells were infected with 1 × 10^8^ transduction units of recombinant lentivirus treated with 8 μg/mL polybrene (Sigma‐Aldrich, Poole, UK).

### Western Blotting and Immunoprecipitation

For Western blotting and immunoprecipitation, we referred to previous experimental methods as follows 19. Electrophoretic analysis was performed using 40 or 60 μg of total protein extracts on a 15% sodium dodecyl sulfate polyacrylamide gel. For immunoprecipitation, 1–1.5 mg of total protein from cell lysates was incubated with anti‐Flag antibody (1:2,000, F1804, Sigma‐Aldrich, Poole, UK) for 4 h at 4 °C and then with protein A/G‐Sepharose beads (Beijing Zhonghua Jinqiao, Beijing, China) for 2 h at 4 °C, separated on gels and transferred to polyvinylidene difluoride membranes (Millipore). The membranes were then analyzed using primary antibodies, including KRAS (1:1,000, sc‐30, Santa Cruz Biotechnology), Myc (1:2,000, sc‐40, Santa Cruz Biotechnology), HA (1:2,000, sc‐57592, Santa Cruz Biotechnology), Flag (1:2,000, F1804, Sigma‐Aldrich, Poole, UK), phosphorylated ERK1/2 (pERK1/2; 1:2,000, 4348, Cell Signaling), ERK1/2 (1:2,000, 8544, Cell Signaling Technology), and β‐actin (1:2,000, sc‐47778, Santa Cruz Biotechnology), and then with secondary antibodies (Maixin Biotechnology, Fujian, China). Finally, the proteins on the membranes were revealed using Western blotting ECL substrates (Pierce). PANC‐1 or HEK293T cells transfected with different plasmids were maintained in medium mixed with 100 nmol/L MG‐132 or DMSO or 50 μg/mL cycloheximide (Sigma‐Aldrich, Poole, UK).

### Quantitative Real‐time Reverse Transcription PCR

Total RNA was extracted from the cells with TRIzol reagent (Invitrogen). Real‐time RT‐PCR assays were conducted to detect the relative levels of *KRAS*, and the mRNA level of glyceraldehyde‐3‐phosphate dehydrogenase (*Gapdh*) was set as the internal standard reference. PCR cycles were performed as follows: initial denaturation at 95 °C for 5 min followed by 36 cycles at 95 °C for 20 s, 52 °C for 20 s, and 72 °C for 30 s. We used the 2−ΔΔCt method to calculate the relative quantities of mRNA. The PCR primers were selected based on previous literature 12.

### CCK‐8 Growth Curve Assays

PANC‐1 cells transfected with the various plasmids were plated in a 96‐well plate at a density of 1,000 cells per well. The cells were maintained in culture medium with 10% CCK‐8 (Dojindo Laboratories, Kumamoto, Japan). The plate was incubated at 37 °C for 1.5 h until visible color changes were observed. Each sample was tested in triplicate. Proliferation rates were determined after 24, 48, 72, 96, and 120 h. The absorbance at 450 nm was measured using a Vmax microplate spectrophotometer (Molecular Devices).

### Colony Formation on Soft Agar

PANC‐1 cells infected with pLenti‐AZ‐RFP, pLenti‐RC‐ODC‐GFP, or the control lentivirus were plated for colony formation in 6‐well plates at a density of 3,000 cells per well. The plates were solidified with agar (0.5% agar in culture medium) on the bottom of the wells in advance. Each sample was tested in triplicate. Colonies were allowed to grow at 37 °C in a 5% CO_2_ atmosphere and were observed under a fluorescence microscope (Leica, Solms, Germany) and photographed every three days. After 14 days, the numbers of colonies were counted.

### Tumorigenesis Experiment in Nude Mice

The tumorigenicity of PANC‐1 cells in vivo was assessed in 6‐week‐old male BALB/c nude mice. The mice were randomly divided into four groups of six mice each. We injected PANC‐1 cells stably infected with pLenti6.3‐RC‐ODC‐GFP, pLenti6.3‐AZ‐RFP, or pLenti6.3‐AZ‐RFP combined with pLenti6.3‐RC‐ODC‐GFP into the dorsal subcutaneous region of the mice. By the 10th day, tumor masses could be visualized, and the tumor volumes were then measured every 3 days and recorded as (length) × (width)^2^/2. The mice were euthanized when the maximum diameter of the tumors reached 15 mm. The tumors were subsequently soaked in 10% neutral formalin buffer. All operations were carried out after approval was received from the Animal Experimental Ethics Committee of Zhengzhou University.

### Immunohistochemistry

The tumor tissues of the nude mice were fixed and then embedded in paraffin. Immunohistochemistry was performed (anti‐KRAS, sc‐30, 1:100, Santa Cruz Biotechnology and Ki‐67, sc‐23900, 1:100, Santa Cruz Biotechnology). Tissue sections incubated with normal IgG (Beijing Zhonghua Jinqiao Biotechnology Co., Ltd.) were used as the negative control group. Finally, the sections were analyzed and photographed.

### Statistical Analysis

The statistical analysis was performed using SPSS 24.0 software. The data from every experiment were recorded as the means ± SEs. Student's *t* test was used to compare the differences between the experimental and control groups. Values of *P* < 0.05 indicate significant differences.

## RESULTS

### Generation of a Reconstructed Chimeric RC‐ODC Protein Molecule to Target the KRAS Oncoprotein

**Figure 1:**
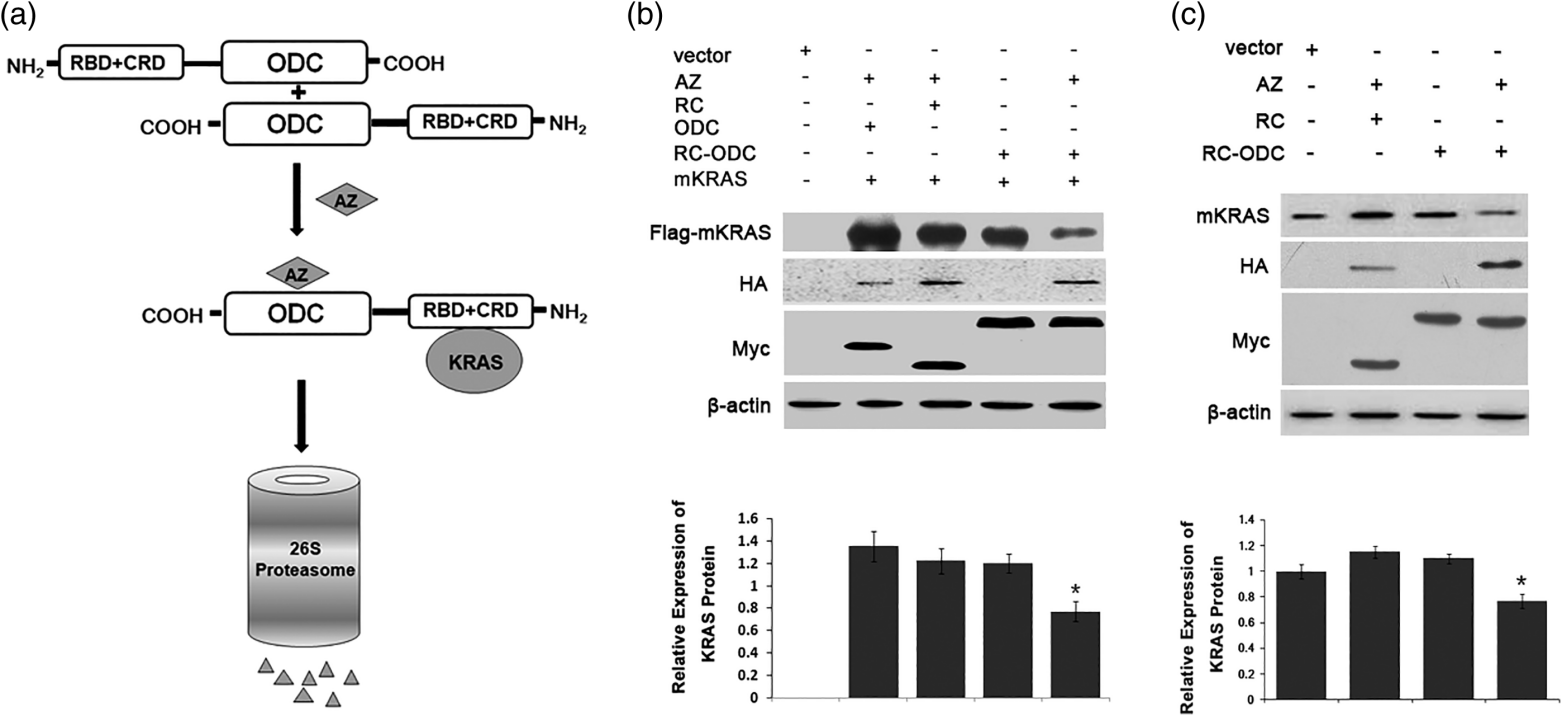
Diagram of AZ‐mediated destruction of target proteins by chimeric proteins ODC and degradation of the KRAS oncoprotein after co‐transfection of RC‐ODC and AZ in human HEK293T cells and the pancreatic cancer cell line PANC‐1. (a) Diagrammatic presentation of the structure of the fusion protein RC‐ODC. The engineered RC‐ODC fusion protein comprises the KRAS‐binding domain RBD + CRD from RAF‐1 fused to the amino terminal and the full‐length ODC at the carboxyl terminus. Homodimers of RC‐ODC are degraded by the 26S proteasome in an AZ stimulation‐dependent manner. (b) RC‐ODC plasmid was co‐transfected with mutant KRAS plasmid and/or AZ plasmid into HEK 293T cells, and the level of the exogenous KRAS protein with the Flag tag was detected 48 h post‐transfection to assess the influence of RC‐ODC and AZ on the KRAS oncoprotein. Ectopic co‐expression of RC‐ODC and AZ notably decreased the KRAS oncoprotein levels (*P* < 0.05). (c) The level of endogenous KRAS oncoprotein in PANC‐1 cells was detected 48 h post‐transfection to assess the influence of RC‐ODC and AZ on the KRAS oncoprotein. The ectopic co‐expression of RC‐ODC and AZ notably decreased the KRAS oncoprotein levels (*P* < 0.05).

We generated a reconstructed fusion protein targeting the mutant KRAS oncoprotein to effectively degrade KRAS via the ODC/AZ system (Fig. 1a). The fragments “RBD and CRD” from RAF‐1, which can bind with mutant KRAS, were selected as the “binding domains” of the reconstructed fusion protein (19. The two domains were fused with the full‐length ODC at the amino terminus of ODC, thus exposing the carboxyl terminus of ODC because the carboxyl terminus of ODC carries the key site for the interaction with proteasomes, which occurs in an ubiquitin‐independent manner. The fusion protein was named Myc‐tagged (RBD‐CRD)^RAF‐1^‐ODC (“RC‐ODC” for short). We co‐transfected RC‐ODC, AZ, and mutant KRAS plasmids into human HEK 293T cells to assess the effect of the RC‐ODC/AZ system on changes in the exogeneous mutant KRAS oncoprotein levels (Fig. 1b). According to Western blotting data, the level of the mutant KRAS oncoprotein in this cell line decreased in the presence of both RC‐ODC and AZ compared with that in the controls (*P* < 0.05). In a previous study, we detected multiple pancreatic cancer cell lines and confirmed that PANC‐1 cells (*KRAS* ^G12D^) had the highest expression level of the KRAS oncoprotein. The level of endogenous KRAS oncoprotein in PANC‐1 cells was then detected 48 h after co‐transfection of RC‐ODC and AZ. As shown in Fig. 1c, the combination of ectopically expressed RC‐ODC and AZ significantly downregulated the level of the KRAS oncoprotein compared with that in the controls (*P* < 0.05). Before cotransfection, we also made it clear the normal expression of Myc‐RC‐ODC, Myc‐ODC, and HA‐AZ in HEK293T or PANC‐1 cells just as shown in Fig. 1b,c.

### Detection of the AZ‐based System for KRAS Oncoprotein Degradation by RC‐ODC

**Figure 2:**
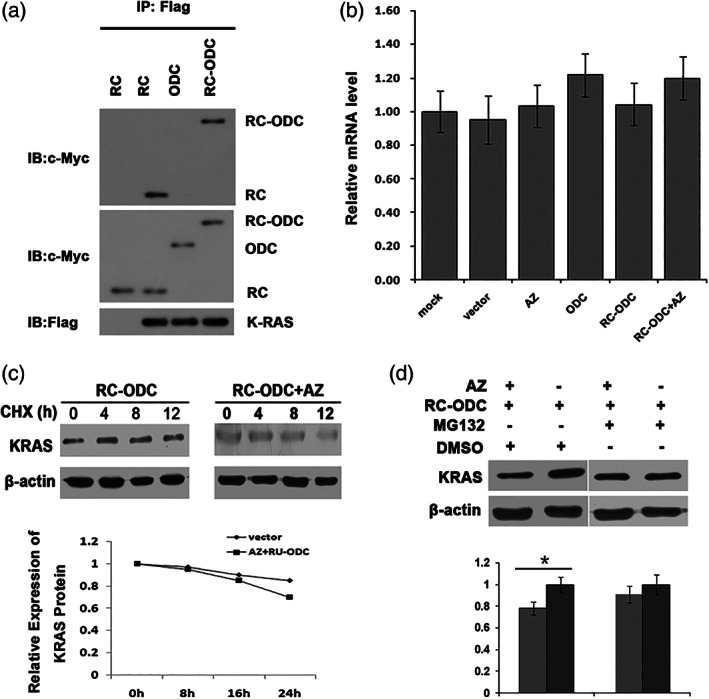
Detection of an AZ‐based system for KRAS oncoprotein degradation by RC‐ODC. (a) Mutant KRAS plasmid with the Flag tag, RC, ODC, and RC‐ODC with the Myc tag were co‐transfected into human PANC‐1 cells, and the fusion protein RC‐ODC co‐immunoprecipitated with mutant KRAS as efficiently as RC. (b) RT‐PCR was used to detect the level of *KRAS* mRNA in cells co‐transfected with AZ and RC‐ODC. No significant differences were observed among the groups (*P* > 0.05). (c) After treatment with the protein synthesis inhibitor cycloheximide (50 ug/mL), the exogenous KRAS oncoprotein level decreased more rapidly in human HEK 293T cells co‐transfected with RC‐ODC and AZ than in the controls. (d) The stability of the KRAS oncoprotein was evidently improved in the presence of MG‐132 (100 nmol/L).

We first detected the potential direct combination of KRAS and RC‐ODC by co‐immunoprecipitation, which is crucial for degradation through the ODC/AZ pathway. Flag‐tagged mutant KRAS and Myc‐tagged chimeric RC‐ODC plasmids were co‐transfected into PANC‐1 cells. As shown in Fig. 2a, RC‐ODC co‐immunoprecipitated with exogenous KRAS oncoprotein as efficiently as RC, indicating that the connection of ODC at the carboxyl terminus of the RC domain did not disturb the combination of RC and mutant KRAS. Meanwhile, the data also showed that ODC cannot bind to KRAS, which was more powerful to prove that RC not ODC in RC‐ODC is the binding domain. To further confirm whether the reduction of the KRAS oncoprotein was independent of gene transcription, RT‐PCR was used to assess the changes in the *Kras* mRNA levels after the co‐transfection of AZ and RC‐ODC. We did not discover any obvious differences among the groups (*P* > 0.05, Fig. 2b), implying that the downregulation of the KRAS oncoprotein occurred at the post‐translational level.

To determine whether this post‐translational downregulation of the mutant KRAS oncoprotein relies on proteasome‐dependent turnover, human HEK293T cells were transfected with the mutant KRAS plasmid along with both the RC‐ODC and AZ plasmids. The cells were treated with the protein synthesis inhibitor cycloheximide for various hours. The level of exogenous KRAS oncoprotein in the co‐transfected human HEK293T cells decreased more rapidly compared with that in the controls (Fig. 2c). To further understand whether the 26S proteasome participates in KRAS oncoprotein elimination mediated by RC‐ODC/AZ, we added DMSO or MG‐132 into the culture medium of PANC‐1 cells to selectively inhibit proteasome activity. The level of the KRAS oncoprotein did not decrease significantly in the cells treated with MG‐132 (Fig. 2d) when RC‐ODC and AZ were cotransfected, compared with the control group in which DMSO was added. Based on these results, RC‐ODC may serve as an effective fusion protein that degrades the KRAS oncoprotein in a proteasome‐dependent manner through the ODC/AZ pathway.

### Co‐transfection of AZ and Fusion Protein RC‐ODC Suppressed the Proliferation of PANC‐1 Cells In vitro

**Figure 3:**
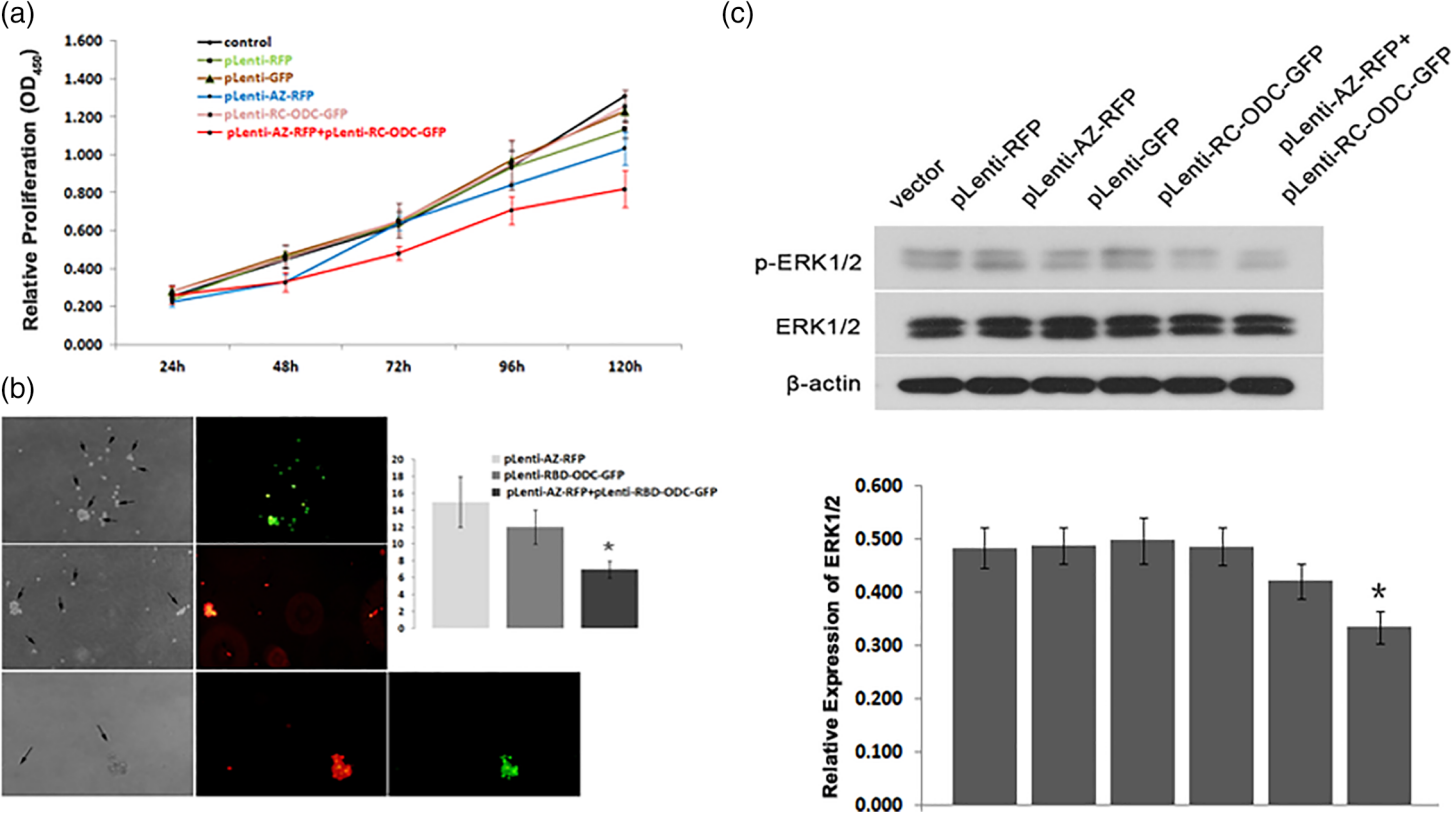
Co‐transfection of RC‐ODC and AZ suppressed the growth of PANC‐1 cells in vitro. (a) PANC‐1 cells co‐infected with lentiviruses expressing RC‐ODC and AZ exhibited lower growth rates in CCK8 assays (*P* < 0.05). (b) The colony formation (just as shown by arrows) ability of PANC‐1 cells co‐infected with lentiviruses expressing RC‐ODC (green) and AZ (red) decreased compared with that of the controls (*P* < 0.05). (c) The co‐transfection of RC‐ODC and AZ significantly decreased the level of pERK1/2, a major downstream effector in the *RAS* signaling transduction pathway, compared with that in the controls.

Mutant *KRAS* has important functions in influencing a variety of malignant characteristics of tumor cells. It has been confirmed that *KRAS* depletion can inhibit pancreatic cancer cell progression. In the present study, we used CCK‐8 growth curves and colony formation assays to evaluate the growth status of transfected PANC‐1 cells. The results showed that PANC‐1 cells co‐infected with lentiviruses carrying AZ and RC‐ODC showed a slower growth rate (*P* < 0.05; Fig. 3a). Furthermore, the co‐expression of RC‐ODC and AZ also markedly suppressed the colony formation of PANC‐1 cells (*P* < 0.05; Fig. 3b). Previous studies have shown that Ras/Raf/MEK is an important signal transduction pathway for the proliferation and survival of pancreatic cancer cells; thus, the level of activated pERK1/2 was detected to investigate whether the co‐transfection of RC‐ODC and AZ affected cell survival through this pathway. As shown in Fig. 3c, compared with the controls, the co‐transfection of RC‐ODC and AZ significantly downregulated the expression of activated pERK1/2. Taken together, these data clearly demonstrated the biological inhibitory effect of the RC‐ODC/AZ system on the proliferation of PANC‐1 cells in vitro.

### Co‐transfection of RC‐ODC and AZ Suppressed Tumorigenicity In Vivo

**Figure 4:**
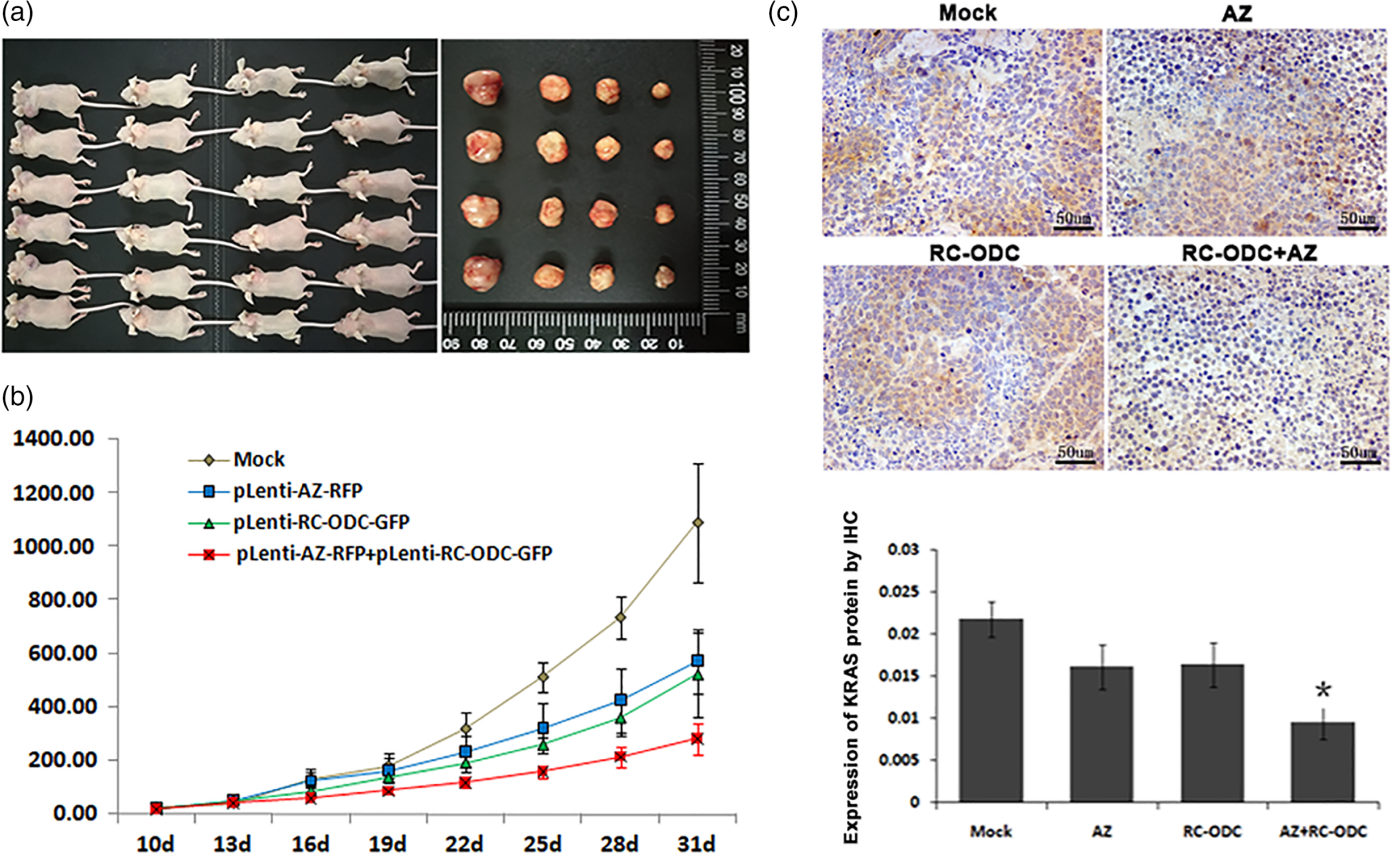
Co‐expression of RC‐ODC and AZ suppressed tumorigenicity in vivo. (a) The gross morphology of the tumors from the four groups of six mice each is shown. (b) A significant difference in tumor volume was detected among the groups starting on the 19th day (*P* < 0.05). (c) Tumor tissues were subjected to immunostaining analysis of the KRAS protein, and the group co‐treated with RC‐ODC and AZ showed relatively weaker cytoplasmic expression of KRAS compared with the mock and control groups. The quantitative analysis of KRAS protein levels is also shown.

Finally, we subcutaneously injected PANC‐1 cells stably infected with RC‐ODC, AZ, or the control vector into nude mice and compared the growth status of the tumors in vivo to assess the effects of the co‐transfection of RC‐ODC and AZ on the tumorigenicity of PANC‐1 cells in vivo. The final tumor masses were arranged according to different groups, as shown in Fig. 4a. We observed an obvious difference in the sizes of tumors among the groups starting on the 19th day (Fig. 4b, *P* < 0.05). As demonstrated through immunohistochemistry, the positive expression of the KRAS oncoprotein in the cytoplasm of tumor cells was relatively weak in the tumors derived from the RC‐ODC/AZ group (Fig. 4c).

## DISCUSSION

Pancreatic cancer is a type of digestive system tumor with a high degree of malignancy, and at the present stage, its early diagnosis and clinical treatment are difficult. In recent years, the morbidity and mortality of this cancer have increased significantly, and the 5‐year survival rate is less than 1% 31. New valid methods are urgently needed for the treatment of pancreatic cancer. An increasing number of studies have focused on gene targeting therapy for pancreatic cancer patients.

Strategies to silence genes at the transcriptional level, including ASO or RNAi, can “knockdown” or “knockout” genes based on the principle of accurate complementary base pairing 32. However, these gene silencing methods are not always effective, either because the elimination of protein products is indirect and dependent on the inherent turnover of the proteins or functional complementarity among multigene families 33, 34. Direct interference with gene expression at the post‐translational level is a promising strategy for exploring the function of a gene or protein and may facilitate the development of novel and effective targeting therapeutics. However, the techniques used for ubiquitin‐dependent protein degradation through the UPP had certain restrictions in previous studies due to the compatibility of the combination of reconstructed fusion E3 ligases and the targeted protein substrate, and the activity of E3 ligases in determining whether ubiquitin molecules could be effectively added onto the targeted protein, which is a critical step for successful degradation. Therefore, the experimental operation is relatively complicated and technically complex.

Previous studies have reported that ODC and protein substrate compounds can be recognized and degraded by the 26S proteasome without ubiquitination of the protein substrate. In this process, the requirement of protein substrate ubiquitination, which is pivotal for recognition by the proteasome, is replaced by an interaction between ODC and AZ, a polyamine‐induced protein 29. The high‐affinity combination of AZ and ODC leads to the formation of ODC/AZ heterodimers. Upon direct binding with AZ, the spatial configuration of ODC is apparently changed, and the 423–461 amino acid sequences at the carboxyl terminal then undergo unfolding, providing a recognition signal to the 26S proteasome 30. In fact, AZ stimulates elimination of the ODC and protein substrate complex by enhancing the interaction of ODC with the 26S proteasome, accelerating their elimination by 50‐ to 100‐fold 26. One important advantage of the ODC/AZ system over most ubiquitin‐based degradation processes is that ubiquitination of the protein substrate at the post‐translational level is not necessary, offering a more direct and more rapid means of delivering the protein substrate to the 26S proteasome for degradation.

In the present study, a novel engineered fusion protein named RC‐ODC, which connected RBD + CRD derived from the RAF‐1 protein, the binding domain of KRAS, with the full‐length ODC, was designed. We used the RC‐ODC/AZ system to “knockdown” the exogenous or endogenous KRAS oncoprotein at the post‐translational level in human HEK293T cells and the pancreatic cancer cell line PANC‐1, and our data imply the tractability of KRAS as a target degraded by the ODC/AZ pathway through the 26S proteasome without ubiquitination.

As shown in our study, the linkage of the RBD + CRD domain of RAF‐1 to the amino terminus of ODC did not disrupt the interaction of RBD + ODC and the KRAS oncoprotein. Furthermore, expression of the RBD + CRD domain at the amino terminus of the fusion protein RC‐ODC exposed the proteasome binding site at the carboxyl terminus of ODC, which is essential for smooth entry of the protein complex into the proteasome for degradation in a ubiquitin‐independent manner 29, 30. In fact, in the present study, we also observed a slight downregulation of the KRAS oncoprotein after the transfection of RC‐ODC alone, and the decrease in the KRAS oncoprotein level in HEK293T and PANC‐1 cells transfected with RC‐ODC was obviously induced upon AZ co‐expression. Therefore, we speculated that efficient degradation of the KRAS oncoprotein by the 26S proteasome through the ODC/AZ pathway requires AZ as a cofactor, which resembles the wild‐type ODC 35.

However, one problem about the ODC/AZ pathway is that the expression of the chimeric protein ODC or AZ may destroy the polyamine balance in cells, resulting in some abnormal phenomena, and may artifactually influence cell growth. In fact, we did not observe any obvious differences between the RC‐ODC‐ and/or AZ‐transfected groups and the control groups, implying that the ODC/AZ pathway was not particularly sensitive to overexpression of AZ or/and the fusion protein ODC in the pancreatic cancer cell line, at least in PANC‐1 cells. Certainly, our results must be confirmed in other pancreatic cancer cell lines.

As shown in the results, PANC‐1 cell proliferation was suppressed in vitro after co‐transfection of RC‐ODC and AZ, and this effect was accompanied by downregulation of the KRAS oncoprotein. In addition, we also detected the level of ERK1/2, an important effector downstream of the Ras/Raf/MEK signaling transduction pathway 36, 37, and observed a decrease in the activated ERK1/2 level. The suppression of PANC‐1 cell growth in vitro after the co‐transfection of RC‐ODC and AZ prompted us to explore the possible therapeutic effect of RC‐ODC/AZ in tumor growth inhibition in vivo. Therefore, we performed xenotransplantation of tumor cells into nude mice in this study. The group of nude mice co‐treated with RC‐ODC and AZ showed significant tumor growth inhibition. However, we also observed the significant tumor growth inhibition of groups treated with AZ or RC‐ODC separately, compared with the control. We speculated that it might be the toxic effects of different viral plasmids transfection. Furthermore, according to immunohistochemical staining, the levels of positive KRAS protein expression were lower in the tumor tissues from the co‐treated group than in the mock and control groups. These results indicated that the RC‐ODC/AZ pathway may represent an effective treatment strategy for pancreatic cancer patients by targeting KRAS.

Matsuzawa et al. investigated the ODC/AZ pathway in the degradation of 12 protein molecules 38. However, only five of the 12 proteins were degraded successfully in their study, implying that this strategy is only applicable to some proteins. Some possible explanations for the inability to degrade certain protein substrates include (i) a lack of interactions between protein ligands and their target proteins and (ii) the presence of a rigid spatial structure preventing the target proteins from entering the proteasome 39. Therefore, although the ODC/AZ pathway could provide another more direct and more rapid means of delivering the ODC/ligand complex to the 26S proteasome for degradation, the feasibility and effectiveness of this system must be experimentally determined. New widely applicable methods for protein “knockdown” that act exclusively at the post‐translational level should be explored, and our group is now interested in depleting RAS oncoproteins using a strategy called “Trim‐Away” 40.

In summary, the chimeric fusion protein RC‐ODC generated in the present study downregulated the level of the KRAS oncoprotein and led to the suppression of PANC‐1 cell proliferation in vitro and in vivo. Compared with the ubiquitin‐dependent degradation of the KRAS oncoprotein, the targeted “knockdown” of KRAS at the post‐translational level through the ODC/AZ pathway (we also call it “proteasome‐dependent”) is similarly effective, which may represent an additional valid therapeutic strategy in pancreatic cancers.

## CONFLICT OF INTEREST

All of the authors have no conflicts of interest with the contents of this article to declare.
